# Neuromodulation of Olfactory Sensitivity in the Peripheral Olfactory Organs of the American Cockroach, *Periplaneta americana*

**DOI:** 10.1371/journal.pone.0081361

**Published:** 2013-11-14

**Authors:** Je Won Jung, Jin-Hee Kim, Rita Pfeiffer, Young-Joon Ahn, Terry L. Page, Hyung Wook Kwon

**Affiliations:** 1 WCU Biomodulation Major, Department of Agricultural Biotechnology, College of Agriculture & Life Sciences, Seoul, Republic of Korea; 2 Department of Biological Sciences, Vanderbilt University, Nashville, Tennessee, United States of America; University of Houston, United States of America

## Abstract

Olfactory sensitivity exhibits daily fluctuations. Several studies have suggested that the olfactory system in insects is modulated by both biogenic amines and neuropeptides. However, molecular and neural mechanisms underlying olfactory modulation in the periphery remain unclear since neuronal circuits regulating olfactory sensitivity have not been identified. Here, we investigated the structure and function of these signaling pathways in the peripheral olfactory system of the American cockroach, *Periplaneta americana*, utilizing *in situ* hybridization, qRT-PCR, and electrophysiological approaches. We showed that tachykinin was co-localized with the octopamine receptor in antennal neurons located near the antennal nerves. In addition, the tachykinin receptor was found to be expressed in most of the olfactory receptor neurons in antennae. Functionally, the effects of direct injection of tachykinin peptides, dsRNAs of tachykinin, tachykinin receptors, and octopamine receptors provided further support for the view that both octopamine and tachykinin modulate olfactory sensitivity. Taken together, these findings demonstrated that octopamine and tachykinin in antennal neurons are olfactory regulators in the periphery. We propose here the hypothesis that octopamine released from neurons in the brain regulates the release of tachykinin from the octopamine receptor neurons in antennae, which in turn modulates the olfactory sensitivity of olfactory receptor neurons, which house tachykinin receptors.

## Introduction

Olfaction is an important sensory modality for social interactions, perception, and efficient orientation to food sources in most animals [1]. Moreover, olfactory neural processing is closely related to the physiological state of the organisms [2-4]. There is substantial evidence that numerous types of neurotransmitters modulate the central and peripheral steps of odor detection in most animals [5-7]. While this relationship has been widely known for several years, the molecular neural mechanism underlying the alteration of the peripheral olfactory sensitivity induced by the neurotransmitters are not yet understood. 

Available evidence suggests that modulation of olfactory sensitivity is controlled by both neuropeptides and biogenic amines [6,8,9]. Among them, octopamine (OA) is known as a neurotransmitter, a neuromodulator, and a neurohormone in insects [10]. OA is released into the antennal heart of the cockroach [11], and the injection of OA can either decrease or enhance olfactory responses depending on the stimulus [12,13]. While the molecular mechanisms underlying the role of the OA in olfactory modulation are not known, it seems to have several roles on the regulation. In addition to OA, it is likely that certain neuropeptides are also employed as neuromodulators in insect olfactory systems. One neuropeptide of particular interest is tachykinin (TK) [14]. Several isoforms of TK peptides have been shown to be expressed in the central and peripheral nervous systems [15-17]. A recent study found that flies deficient with tachykinin-related peptides exhibited a decrease of sensitivity for odor perception, implicating their significant role of neural modulation in olfactory systems [18]. In addition, it has been reported that the *Drosophila* tachykinin receptor (DTKR), expressed in axon terminals of olfactory receptor neurons (ORNs), is modulated by TK released from local interneurons (LNs) of antennal lobes (ALs) and inhibits the olfactory responses of ORNs [19]. However, most research has focused on the central nervous system (CNS), and thus information on how the function of peripheral olfactory systems can be regulated is largely unknown. 

In this regard, we have employed *in situ* hybridization, immunostaining, and quantitative real-time polymerase chain reaction (qRT-PCR) methods to investigate the anatomical organization of neuronal circuits governing the alteration of olfactory sensitivity by both OA and TK signaling in the cockroach antenna. In addition, we report the observations on the physiological effects of tachykinin, octopamine, and octopamine receptors on olfactory sensitivity in the antennae of *Periplaneta americana*. Our current data demonstrate that the molecular machinery involved in the modulation of olfactory sensitivity exists in the peripheral olfactory organ. 

## Materials and Methods

### Animals and tissue preparation

*P. americana* were raised under a light cycle of 12:12 hours light and a dark (L/D) cycle at 26±1°C as described [20]. Intact adult male cockroaches were used for qRT-PCR and electrophysiological recordings. Animals with external damage such as missing antennal segments were discarded for tissue preparation. Male cockroaches were anesthetized by chilling on ice before dissection after which antennae were immediately placed on dry ice. Further experimental processing of the tissue for histology is described below. 

### Gene Cloning of octopamine receptor (*Pa*OA1), tachykinin (*Pa*TK), and tachykinin receptor (*Pa*TKR) and RNA probes

**Figure 1:**
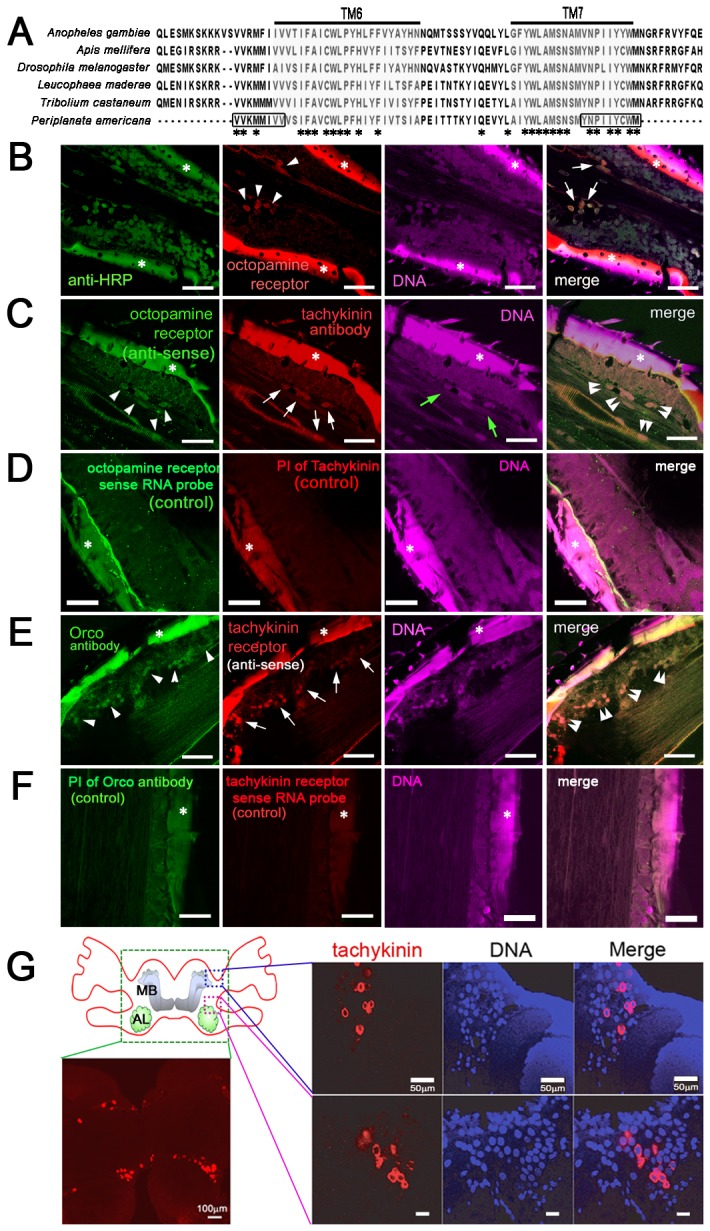
Localization of octopamine receptors, tachykinin, and tachykinin receptors in the antennae of *P. americana*. (**A**) Partial amino acid sequence of tachykinin receptors of *Periplaneta americana* (*Pa*TKR) and its alignment with other insect tachykinin receptors present in mosquito (*Aedes aegypti*), honey bee (*Apis mellifera*), fruit fly (*Drosophila melanogaster*), red flour beetle (*Tribolium castaneum*), and Madeira cockroach (*Leucophaea maderae*). Transmembrane regions 6 and 7 are shaded in gray. Amino acid residues selected for gene cloning were indicated by boxes. (**B**) *In* *situ* hybridization with antisense RNA probes of octopamine receptor (red), where octopamine receptors (arrowheads) were co-localized with neuronal marker anti-HRP antibody (arrows). (**C**) Expression of tachykinin (arrows) in octopamine receptor neurons (green, arrowheads) were co-localized (double arrowheads). Green arrows indicate antennal nerves. (**D**) Control of *in* *situ* hybridization using sense RNA probes of octopamine receptor and pre-immune serum of tachykinin. (**E**) Tachykinin receptor (arrows) of *P. americana* was co-localized (double arrowheads) with odorant receptor co-receptor (Orco, arrowheads) in olfactory sensory neurons of the antennae. (F) Control of *in* *situ* hybridization using sense RNA probes of tachykinin receptor and pre-immune serum of Orco. Scale bars demonstrate 20 μm. (**G**) Immunostaining with tachykinin antibody in brain tissue of cockroach. A schematic diagram of the cockroach brain showed mushroom body (MB) and antennal lobes (AL) where tachykinin (red) was mainly localized. Images demonstrate tachykinin staining in the brain area indicated with a dotted box. Cell bodies were stained with DAPI (blue). Scale bars demonstrate 20 μm unless indicated. Asterisks demonstrate the autofluorescence from antennal cuticle.

Total RNA was extracted from three male *P. americana* antennae and brains using the Qiagen RNasy kit by manufacturer’s instruction (Qiagen, Valencia, CA, USA), after which RQ1 RNase-free DNase I (Promega, Madison, WI, USA) was treated by manufacturer’s instruction. Reverse transcription procedures were carried out as described previously [21]. In order to clone PaTKR mRNA, TKR sequences of *L. maderae* [22] (CAC36957.1), *Tribolium castaneum* (XP_970102.1), *Anopheles gambiae* (XP_312088.3), *Apis mellifera* (XP_395081), and *D. melanogaster* (AAA28722.1) were used for multiple alignment for conserved domain of the transmembrane protein regions (Figure 1A). Partial cDNA clones for *Pa*OA1 and *Pa*TKR were generated using specific primer sets as shown in Table S1. These genes were inserted into a pGEM-T vector (Promega), after which RNA probes were made using a DIG RNA labeling mix as manufacturer’s instructions (Roche, Indianapolis, IN). 

### Quantitative real-time PCR (qRT-PCR)

Adult male cockroaches were isolated in a plastic cage and were provided with food and water *ad lib*. cDNA synthesis was conducted as described above. qRT-PCR was carried out with the StepOnePlus (Applied Biosystems, Foster City, CA, USA) using SYBR green qPCR Master Mix (Fermentas, Ontario, Canada). Primer information for qPT-PCR was described in Table S1. Quantitative analysis was employed by StepOne plus Software V. 2.0 (Applied Biosystems). Results were normalized to a validated control gene, *actin*, using the ΔΔCt method [23]. pQE30 vector sequence was used as a control. Gene-specific primers used for qRT-PCR were as follows: PaOA1 (AY333178.1): Forward primer (FP): 5'-CTCTTCTGGCTGGGCTATTG-3', Reverse Primer (RP): 5'-TCCTTGCTAAAGAGGGCG TA-3'; PaTK: FP: 5'-GCAAGAAGGCACCATCAGC-3', RP: 5'-ATGCCCATAAACCCGG AAC-3', PaAct (AY116670.1): FP: 5'-GCTATCCAGGCTGTGCTTTC-3', RP: 5'-ACCGGAA TCCAGCACAATAC-3'

### *In situ* Hybridization, immunostaining, and imaging

Antennae and heads of male *Periplaneta americana* were fixed with 4% paraformaldehyde (PFA) solution overnight, after which antennae were cut at 3-5mm long and brains were dissected out. Tissues were then washed with PBS buffer (pH 7.4), followed by dehydration and rehydration using a series of ethanol from 25%~100%. Tissues were then hybridized with a hybridization solution containing Dig-labeled RNA probes for 20 hrs at 58°C. After several washes with PBS buffer containing 0.2% Tween 20, tissues were incubated with peroxidase (POD)-conjugated anti-DIG antibodies (Roche, Indianapolis, IN) in a blocking reagent (Roche) overnight at 4°C, followed by signal visualization process using a Tyramid signal amplification (TSA) kit by manufacturer’s instruction (PerkinElmer, Waltham, MA, USA). After in situ hybridization, immunostaining using several different antibodies were performed as described previously [21]. Tissue preparations after *in situ* hybridization were subsequently incubated with rabbit anti-tachykinin polyclonal antibodies (1:2000 in PBS with 0.1% Tween 20, PBSTw) against *Locusta migratoria* tachykinin [24] (gifted from Dr. Yoon-Seong Park at Kansas State University) or anti-DmOrco polyclonal antibody (gifted from Dr. Leslie Vosshall at Rockefeller University) to immuno-localize Orco of *P. americana* at 4°C overnight. After 3-4 times washing with PBSTw, anti-rabbit secondary antibodies conjugated with cy3 fluorophore were incubated overnight at 4°C. Cell and neuronal staining was conducted with TOTO-3 (Invitrogen) and anti-horseradish peroxidase (HRP) antibody (Jackson ImmunoResearch Laboratories, West Grove, PA, USA), respectively. 

Stained tissue preparations were dehydrated with a series of ethanol and 100% acetone to embed into Spurr’s epoxy resin [21]. Resin-embedded tissues were incubated at 60°C oven overnight and sectioned at 20μm thickness by using an automatic sliding microtome (HM 355S; Microm, Thermo Scientific). Sectioned tissues were mounted with Serva fluoromount (Crescent Chemical Co). Images were captured with a LSM 700 confocal microscope (Zeiss, Thornwood, NY) and were processed with ZEN 2009 software. Gene-specific primers for preparing RNA probes were as follows: PaOA1 (AY333178.1): FP: 5’-CAACAGCTCCAAGAAGTCCAG-3’, RP: 5’-GCTGTCCTCTCCTACCGAGTT-3’; PaTK (AY766012.1): FP: 5’-CCCCATCACACAACAAGAGTT-3’, RP: 5’-CCCTCCATCTC TGAGTCCTTT-3’; PaTKR- FP: 5’-GGGTAGTGAAGATGATGATTGTGGTGG-3’, RP: 5’-TCATCCAACAGTAGATGATGGGATTGTAC-3’

### Injection of octopamine, tachykinins, dsRNAs, and octopamine agonist and antagonist

Fifteen tachykinin peptides identified in the cockroach, *P. americana* (*Pa*TKs) [15] were synthesized with 95% purity (Anygen, Gwang-Ju, Korea) (Table S2). dsRNAs for *Pa*OA1, *Pa*TK, *Pa*TKR mRNA as well as pQE30 as a control gene were generated using MEGAscript RNAi kit (Ambion) with specific primer sets (Table S1). For the injection of tachykinins and dsRNAs, 3 μl of each active tachykinin with 60 nmol and 0.5 μl of synthesized dsRNA (5 μg/μl) were injected into the antennal base of intact adult male cockroaches caught at CT11 using a Hamilton micro syringe, needle G30 (Becton Dickinson). For injection experiments of octopamine and TK-dsRNA and octopamine alone, 5 μl of 10mM octopamine were used. Injected cockroaches were kept individually. Control cockroaches were injected with saline (PBS. pH=7.4). 2 μl of octopamine agonist (clonidine) and antagonist (yohimbine) (Sigma-Aldrich) were injected at the concentration of 1M into the same areas of cockroach antennae described above.

### Electroantennogram (EAG)

A male cockroach was restrained with one antenna fixed in an EAG setup, as previously described [25]. The electrical signals were amplified (IDAC4, Syntech, The Netherlands) and processed with EAGPro software (Syntech). Prior to injection of tachykinin and dsRNA, three to five pre-recordings with 5 min intervals for olfactory responses to ethyl acetate (1 sec stimulation), diluted to 10^-2^ (vol/vol) with mineral oil to a final concentration of 10 mM (Sigma-Aldrich), were measured. Five min after injection of tachykinin and dsRNA, 13 to 20 consecutive post-recordings were made to ethyl acetate delivered every 5 min. EAG amplitudes were normalized by dividing a mean amplitude value of pre-recording (before injection) from each olfactory amplitude during post-recordings (after injection). EAG recordings were conducted in the middle of the animal’s subjective day. This is near the peak sensitivity of the circadian rhythm in olfactory response in cockroaches [20,25]. 

### Single sensillum recording (SSR)

Olfactory responses using SSR were measured from single-walled type C sensilla by inserting the tip of glass electrode into the cuticle at the base of the sensillum. Odorant stimulation was the same as in the EAG recording setup described above. Olfactory responses were measured by the number of spikes (action potentials) elicited by ethyl acetate stimulation by deducting spike numbers during 1 sec post-stimulation from 1-sec pre-stimulation. Three to five recordings prior to injection and 13 to 21 consecutive post-injection recordings were analyzed (N=12). Antennal preparation was viewed at 1,000 magnification by a BX-51 microscope (Olympus). Electrical signals elicited by odorant stimulation were imported to a 4-channel IDAC-USB and analyzed with AutoSpike software (Syntech, The Netherlands). Spike sorting and counting by the AutoSpike program were clearly distinguishable according to spike amplitude compared to background noises. The number of spikes, as an indicator of olfactory responses, was calculated as follows: the number of spikes that occurred during the first 1 s after the onset of odor stimulation minus the number of action potentials during the 1 s immediately prior to the onset of the stimulus. .

### Statistical analysis

Gene expression data were analyzed by Student’s *t*-test (SPSS, Version 20, IBM, NY, USA). Comparisons of EAG and SSR responses between pre-injection and post-injection were analyzed using a one-way ANOVA test followed by Bonferroni correction with multiple comparisons (SPSS, Version 20, IBM, NY, USA). Time frames were divided as a group before and after injection during EAG and SSR. Statistical analysis was conducted among these groups.

## Results

### Cloning of octopamine receptor, tachykinin, and tachykinin receptor of *P. americana*

The *P. americana* genome has been reported to contain genes encoding octopamine receptor (*Pa*OA1) and TK (*Pa*TK) neuropeptides [15,26]. We subcloned these genes using gene-specific primer sets as shown in Table S1. In contrast, the tachykinin receptor gene of *P. americana* (*Pa*TKR) was not available in the GenBank. For this reason, the conserved regions of TKR protein sequences from several different species of insects were aligned, especially in transmembrane regions 6 and 7 (TM6 and TM7) (Figure 1A). Specific primer sets were able to clone partial *Pa*TKR gene sequence (192bp) as a novel gene (Table S1). The cloning of the full coding sequence of *Pa*TKR gene remains to be accomplished. 

### Localization of *Pa*OA1, *Pa*TK, and *Pa*TKR mRNA in antennae

*Pa*OA1, *Pa*TK and *Pa*TKR in cockroach antennae were localized by using double-labeling studies with *in situ* hybridization and immunostaining (Figure 1). First, *Pa*OA1 mRNA were localized in the antennal neurons (Figure 1B) compared to control (Figure 1D). Neuronal clusters consisting of 5~6 neurons that expressed *Pa*OA1 mRNA were found in each antennal segment near the area of antennal nerves (arrowheads in Figure 1B and C) where *Pa*OA1 and *Pa*TK (arrows in Figure 1C) were co-localized in the same neurons of the antennae (double arrowheads in Figure 1C). Immunohistochemistry using tachykinin antibody in the brain of the cockroach showed that tachykinin-positive neurons are located in the vicinity of antennal lobe (AL) and mushroom body (MB) calyces (Figure 1G). Double-label studies using an anti-sense RNA probe of the *Pa*TKR and an antiserum to the *D. melanogaster* odorant receptor co-receptor (*Dm*Orco) overlapped in ORNs of the antennae (Figure 1E) when compared to control (Figure 1F). *Dm*Orco gene is well-conserved among different insect species and forms heterodimers with ligand-specific conventional odorant receptors (ORs) in the ORNs, as reported previously [27]. Intriguingly, our results demonstrated that most ORNs (arrowheads in Figure 1E) also expressed the TKRs (arrows and double arrowheads in Figure 1E). 

### Effects of tachykinin (TK) on olfactory sensitivity in antennae

**Figure 2:**
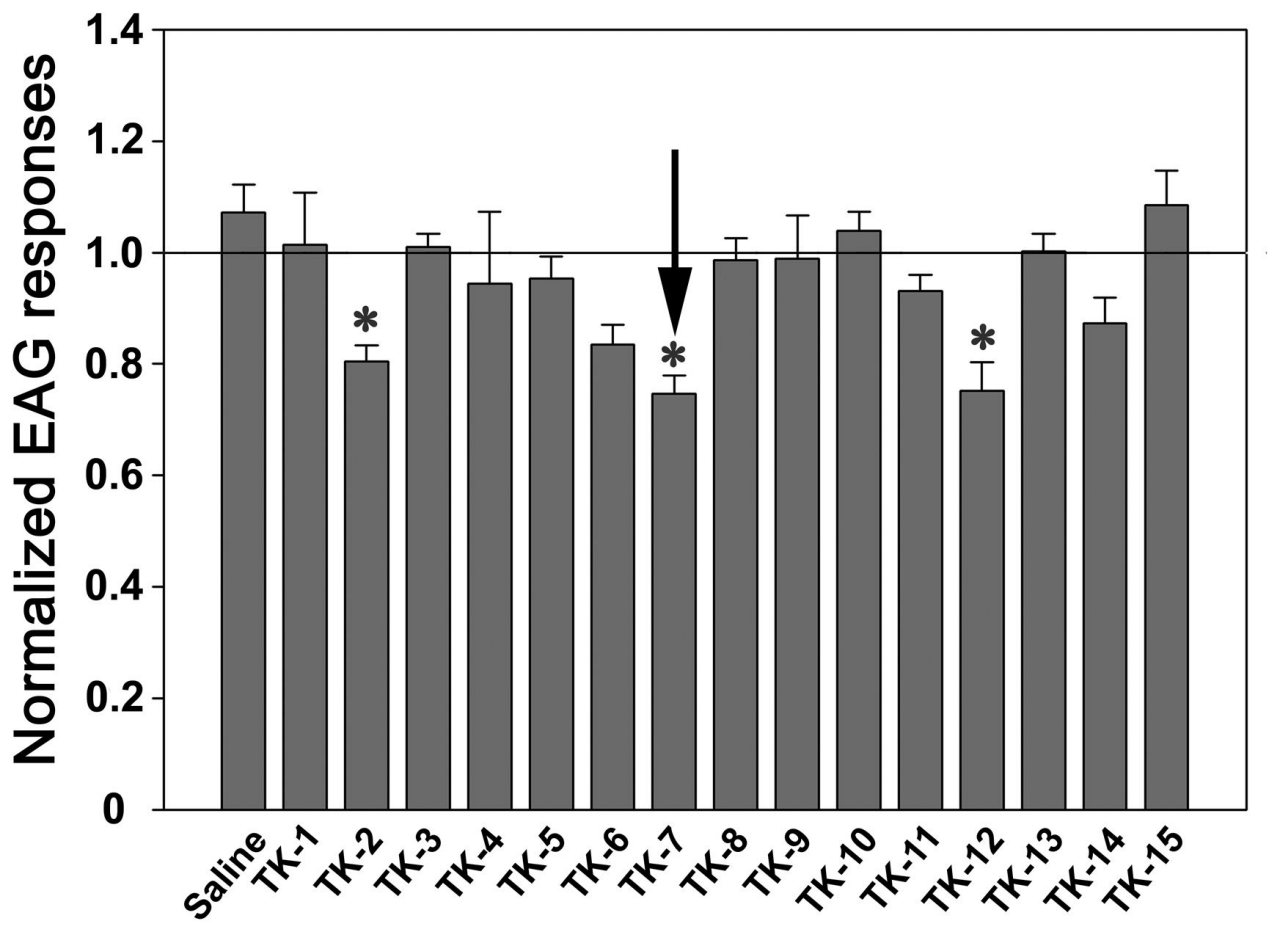
Normalized EAG responses of cockroach antennae to ethyl acetate (10^-2^ dilution) after injection of tachykinin (TK) peptides. EAG amplitudes were decreased after injection of TK-2, -7, and -12, compared to other TK peptides and control saline injection (N=4-8, ANOVA with Bonferroni correction, *=p<0.05). TK-7 (arrow) showed strongest effects on decrease of olfactory responses, which was in turn used for further experiments. Effects of other TKs on EAG responses were not significantly different from saline control injection. Information on synthesized TK peptides was as follows: PaTK1: APSGFLGVR-NH2, PaTK2: APEESPKRAPSGFLGVR-NH2, PaTK3: NGERAPASKKAPSGFLGTR-NH2, PaTK4: APSGFLGTR-NH2, PaTK5: APGSGFMGMR-NH2, PaTK6: APAMGFQGVR-NH2, PaTK7: APASGFFGMR-NH2, PaTK8: VPASGFFGMR-NH2, PaTK9: GPSMGFHGMR-NH2, PaTK10: APSLGFQGMR-NH2, PaTK11: APNMGFMGMR-NH2, PaTK12: MGFMGMR-NH2, PaTK13: GPSVGFFAMR-NH2, PaTK14: APSAGFMGMR-NH2, PaTK15: APSAGFHGMR-NH2.

**Figure 3:**
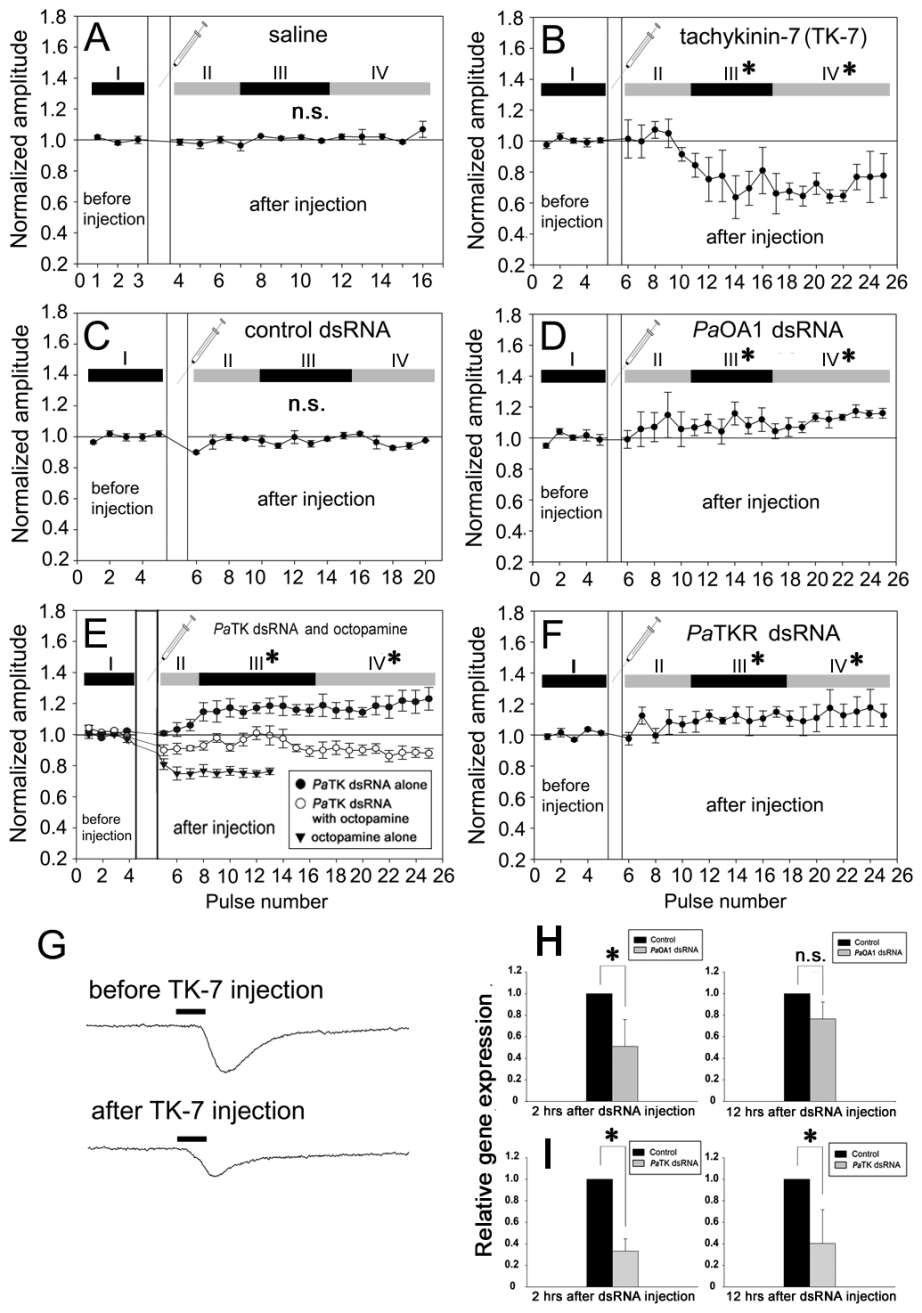
Effects of “before and after injection” of tachykinin, octopamine receptors, tachykinin receptors on olfactory responses (EAG) in antennae and quantitative real-time PCR after dsRNA injection of tachykinin and octopamine receptor. (**A**) There was no significant difference before and after a control injection of saline (N=3, p>0.6). (**B**) Tachykinin-7 (TK-7) injection produced a significant decrease in olfactory responses phase II and phase III after injection compared to before injection (phase I) (N=5, *=p<0.001). (**C**) Injection of control dsRNA from an expression vector (pQE30) did not significantly alter EAG responses (N=5, p>0.7). (**D**) Injection of octopamine receptor (*Pa*OA1) dsRNA elicited significant increase in EAG amplitude in Phase III and IV, which continued to over 2 hours after injection (N=5, p<0.001). (**E**) Three EAG traces showed interaction between octopamine and tachykinin on the modulation of olfactory sensitivity in cockroach antennae. Injection of octopamine itself (inverted triangle) decreased EAG response after injection in Phase III and IV, while TK-dsRNA (filled circle) induced significant increase of EAG amplitude (N=5, p<0.01). Notably, the enhanced effect of TK-dsRNA on EAG responses was eliminated when co-injected with octopamine (empty circle). (**F**) Injection of tachykinin receptor (*Pa*TKR) dsRNA had a significant positive effect on EAG amplitude (N=3, p<0.001). (**G**) Representative EAG traces before and after TK-7 injection, showing that TK-7 decreased EAG responses. (**H**) Relative RNA expression levels of octopamine receptor (*Pa*OA1) in antennae after injection of octopamine receptor dsRNA were strongly reduced at 2 hours after injection but not significantly different 12 hours after injection (Student’s *t*-test: *, P≤0.05). (**I**) Relative RNA expression levels of tachykinin gene (*Pa*TK) in antennae showed significant reduction at 2 and 12 hours after injection. Values depict mean ± SE. One-way ANOVA test followed by Bonferroni correction for multiple comparisons was employed to test the difference in EAG responses, while Student t-test was used to test the difference of gene expression levels. Pulse number indicates the number of ethyl acetate stimulation every five minutes. Phase I depicts EAG responses during “before injection” and phases II, III, and IV indicate EAG responses during “after injection”.

**Figure 4:**
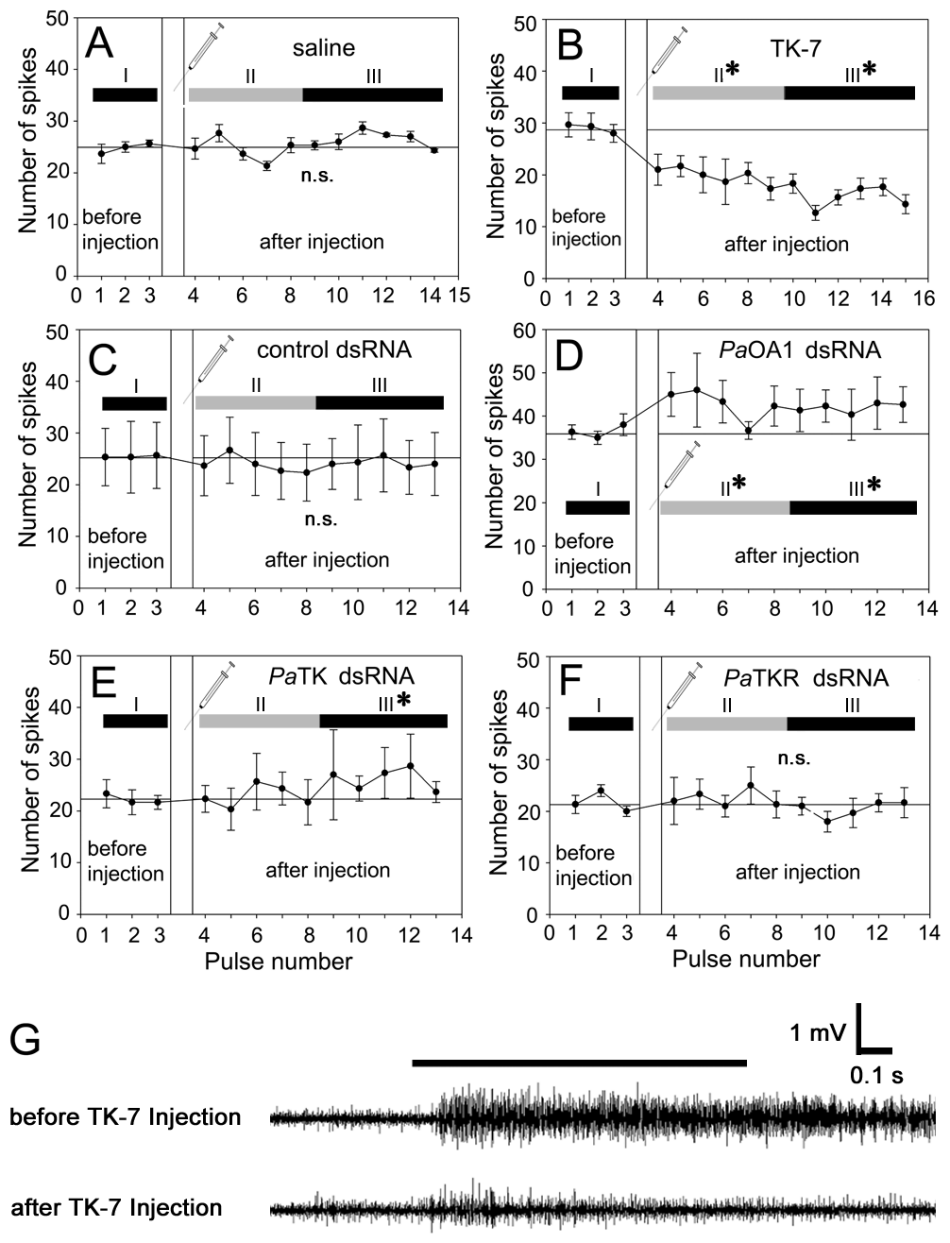
Olfactory responses to ethyl acetate by TK-7 and other dsRNA treatment using single sensillum recording. (**A**) Saline injection caused no significant changes after injection (N=5, p>0.5). (**B**) Application of TK-7 induced a substantial decrease in the spike activities of olfactory receptor neurons (N=4, p<0.001). (**C**) Control non-specific dsRNA did not alter olfactory responses (N=4, p>0.2). (**D**) Octopamine receptor (*Pa*OA1) dsRNA treatment resulted in significant increase in spike numbers (N=5, p<0.01). (**E**) Tachykinin (*Pa*TK) dsRNA injection exhibited no significant changes in olfactory responses at phase II (4~8^th^ pulses) (N=4, p>0.9) but significant increase in phase III (after 9^th^ pulse) (N=4, p<0.02). (**F**) Tachykinin receptor (*Pa*TKR) dsRNA injection did not show significant changes (N=5, p>0.7). (**G**) Representative olfactory response traces by SSR upon TK-7 injection. Significant decrease of spike trains was shown, indicative of decrease of olfactory sensitivity. One-way ANOVA test followed by Bonferroni correction for multiple comparisons was employed for statistical analysis.

In order to better understand the functional roles of TK on olfactory responses in antennae, 15 synthesized active TK peptides were used to identify the effects on olfactory responses in the antennae (see Figure 2 legend). Among them, TK-2, TK-7 and TK-12 peptides decreased the olfactory responses after TK application (Figure 2, p < 0.05), while no detectable differences from control saline and other TKs were found (Figure 2 and Figure 3A, p > 0.6). TK-7 injection elicited the strongest decrease in EAG responses (Figure 2 and Figure 3B and G, p < 0.05). Notably, a significant decrease in EAG responses was observed at 25 min and the greatest reduction in EAG amplitude was shown 45 min after TK-7 injections (Phase II in Figure 3B). At this point there was approximately a 30% reduction in amplitude after TK-7 application (Figure 3B). Furthermore, recording from single trichoid sensilla after TK-7 injection also showed a decrease in the number of action potentials compared to saline injection as control (Figure 4A, B, and G).

### Alteration of olfactory sensitivity by dsRNA

dsRNA of each gene demonstrated that the transcript levels of both *Pa*OA1 and *Pa*TK mRNAs were prominently reduced for 2 hours after injection (Figure 3H and I, p < 0.05). The RNAi effect was noticeable during a 12-hour period after dsRNA injection. dsRNA injections of *Pa*OA1, *Pa*TK, and *Pa*TKR significantly increased EAG amplitudes about 50 minutes after injection (Phase III and IV), compared to “before injection” (Figure 3D, E, and F, p < 0.01, ANOVA with Bonferroni correction test). Olfactory responses in single sensillum levels were also affected by dsRNA injection (Figure 4). The number of action potentials was slightly increased after injection of *Pa*OA1 dsRNA (Figure 4D, p < 0.01). In contrast, although we did not observe a strong increase in the number of spikes overall before and after injection of *Pa*TK dsRNA (Figure 4E, p > 0.2, ANOVA, Bonferroni correction test), we found significant differences in phase III (9~13^th^ pulse numbers), compared to spike numbers in “before injection” (Figure 4E, p <0.02). Next, we tried to test whether antennal olfactory sensitivity might be affected by both tachykinin and octopamine. Obviously, the injection of octopamine alone showed the decrease of olfactory responses (inverted triangles in Figure 3E, N=6, p<0.01). With co-injection of *Pa*TK dsRNA and octopamine, we observed no significant increase or decrease in olfactory responses, compared to “octopamine alone” and “*Pa*TK dsRNA alone” (empty circles in Figure 3E). However, EAG responses after co-injection were significantly different, compared to “before injection” (N=4, p<0.01), indicating that octopamine concentration used in this experiment, 5 μl of 10mM octopamine, were rather high to compensate the effect of *Pa*TK dsRNA. 

**Figure 5:**
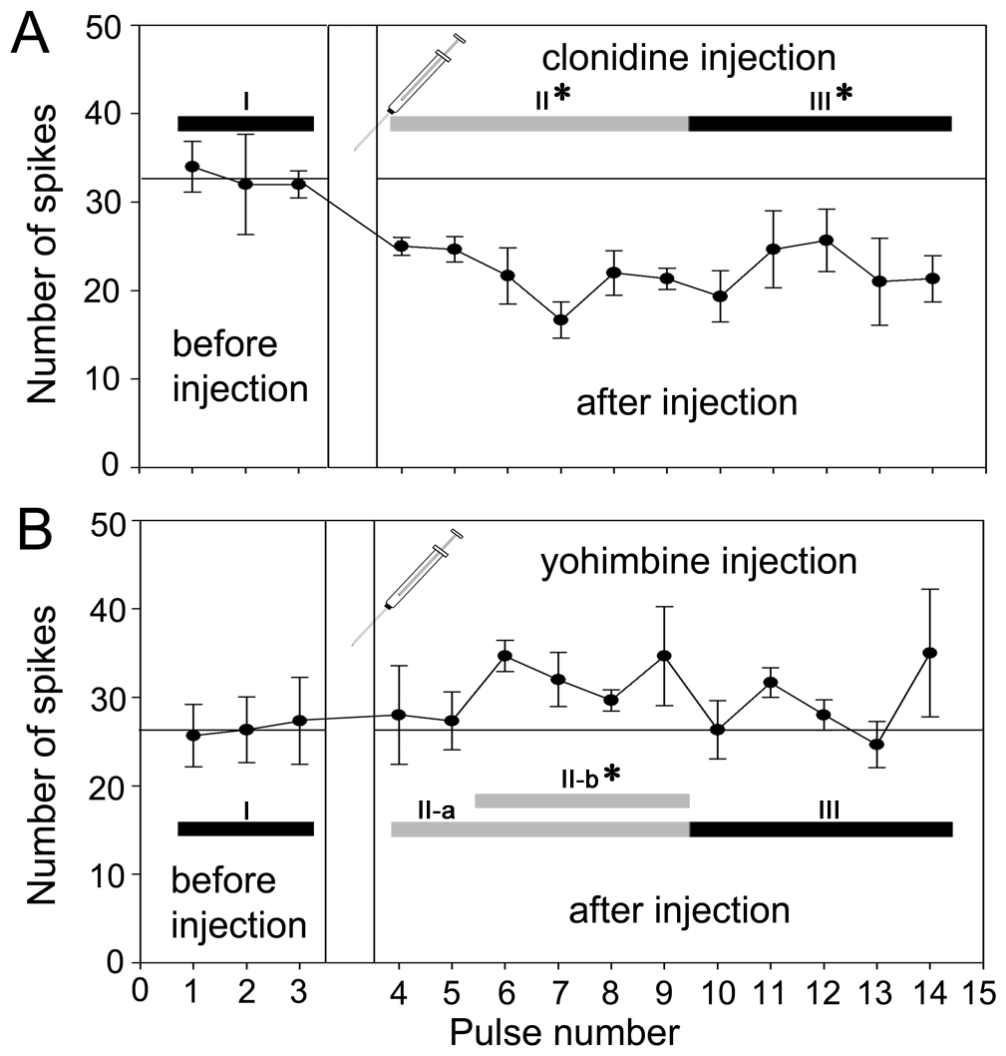
Effect of octopaminergic agonist and antagonist on olfactory responses using single sensillum recordings. (**A**) Injection of clonidine, an octopamine receptor agonist, into the antennae resulted in a drastic decrease in the number of spikes right after injection until the end of the experiment (Phase II and III), compared with “before injection” (Phase I) (N=5, p<0.001). (**B**) Injection of the octopamine receptor antagonist, yohimbine, induced a slight increase in the middle of time phase (II-b, 6^th^~9^th^ pulses), compared with “before injection” (N=5, p<0.001). One-way ANOVA test followed by Bonferroni correction for multiple comparisons was employed for statistical analysis.

Spike numbers upon the dsRNA injection of *Pa*TKR did not show significant differences throughout pulse numbers (Figure 4F, p > 0.7). Spike frequency in response to ethyl acetate stimulation was significantly decreased by clonidine treatment (an octopamine agonist) (Figure 5A, p < 0.001), whereas yohimbine (an octopamine receptor antagonist) elevated the olfactory responses after 6^th^ to 9^th^ odor pulses (phase II-b in Figure 5B, p < 0.001). Other pulses showed no significantly differences. * indicates significant difference of the group compared to before injection. 

## Discussion

A variety of studies have examined the effects of octopamine on olfactory responses in insect antennae. Consistent with our findings here, EAG amplitude has been shown to be decreased by treatment with exogenous octopamine in *P. americana* [28,29]. In contrast, while we found a decrease in action potentials in response to ethyl acetate stimulation, Zhukovskaya and Kapitsky (2006) reported an increase in nerve impulse responses recorded in single sensillar responses to sex pheromone following octopamine injection. Similarly, octopamine was reported to increase the response of pheromone sensitive ORNs in the hawkmoth, *Manduca sexta* [30], the silkmoth, *Antheraea polyphemus* [31], and the cabbage moth, *Mamestra brassicae* [32]. These findings raise the possibility that octopamine may differentially impact the response of sensilla tuned to respond to sex pheromone and those that respond to at least some other odors (e.g., ethyl acetate) and dominate the EAG response. This view is consistent with recent observations in *P. americana* that exogenous octopamine does have differential effects on both the behavioral [12] and olfactory receptor neuron responses [28]. 

In the present study we also find evidence that tachykinin may also play a major role in olfactory modulation. We showed that tachykinin (TK) neuropeptides co-localized with octopamine receptors in antennal neurons in *P. americana*, and may act via tachykinin receptors (TKR) present in ORNs and may act to regulate olfactory sensitivity. These neurotransmitters have previously been found to act as neuromodulators in the visual system by altering the amplitude of responses to light [33]. Substance P, which is a member of TK peptide family in mammals, and octopamine (OA) efferent systems act in concert to increase electroretinogram (ERG) response and regulate the circadian rhythm in sensitivity in visual system of the horseshoe crab, *Limulus polyphemus* [34]. However, the chemical and electrophysiological studies of interactions between OA and substance P are largely unknown. Evidence for OA modulation in the moth *Bombyx mori* demonstrated that OARs were located somewhere near the base of sensilla hairs [35], implying that OA plays an important role in fine tuning of the olfactory responses [28]. Interestingly, cockroach antennae have shown that OA is located in the antennal heart of a neurohemal area, which is secreted from octopaminergic dorsal unpaired median (DUM) neurons in *P. americana* [36]. 

In this regard, our study is the first to demonstrate the fact that OA- and TK-related signaling pathways affect the olfactory regulation of peripheral organs. Co-localization of TK and OARs has suggested the possibility that OA regulates the production or release of TK, which induces the reduction of olfactory responses in ORNs. A previous study has demonstrated that TK-deficient flies exhibit defects in olfactory perception and behaviors responding to specific odorants [18]. Moreover, *Drosophila* tachykinins (DTKs) present in LNs of the ALs, a primary olfactory processing center in insect brains [37], exert a presynaptic inhibitory action to ORNs [19]. This neuronal circuit may regulate olfactory sensitivity to ORNs from AL neurons, modulating innate food searching behaviors under internal and external cues [38]. Given the importance of peripheral olfactory modulation by neuropeptides, food-searching behaviors are also controlled by regulating the insulin signaling in ORNs [6]. It has also been reported that tachykinin receptors and short neuropeptide F receptors (sNPFR) are affected by starvation via insulin signaling in *Drosophila* ORNs [6,39]. These studies also imply that low olfactory responses at a day time enhance the food searching behavior in fruit flies [40]. Likewise, lower olfactory sensitivity has been demonstrated at dusk compared to day time and dawn in a cockroach at which cockroaches habitually show strong food preference [25], thus they may share similar molecular and neural mechanisms modulating olfactory sensitivity. 

Similar peptidergic pathways for the modulation of olfactory sensitivity also exist in vertebrate systems. In Mexican salamander (*Ambystoma mexicanum*), centrifugal peptidergic neurons modulate olfactory responses associated with hunger [41]. Moreover, the tetrapeptide Phe-Met-Arg-Phe-NH_2_ (FMRF-amide) has shown to modulate the neural activities of ORNs in the olfactory epithelium of the mouse and the salamander [42,43]. Recently, several studies has shed light on the olfactory modulation in ORNs in peripheral sites, where acetylcholine is released by micovillar cells of the olfactory epithelium [44] and dopamine is released into the olfactory mucus triggered by exposure to irritants [45]. Another study has found the hormone, leptin and its receptors in olfactory mucosa [7]. Neuropeptide Y (NPY), associated with the regulation of satiety signal of hypothalamus in vertebrates, has also been shown to modulate olfactory responses to a food-related odorant in rat olfactory mucosa by using electro-olfactogram (EOG), where NPY application increase EOG amplitudes in starved rats but not in fed ones [46]. Unlike peripheral modulation, there are ample reports on olfactory sensitivity changes by central mechanisms focused on CNS [19,47-49]. 

**Figure 6:**
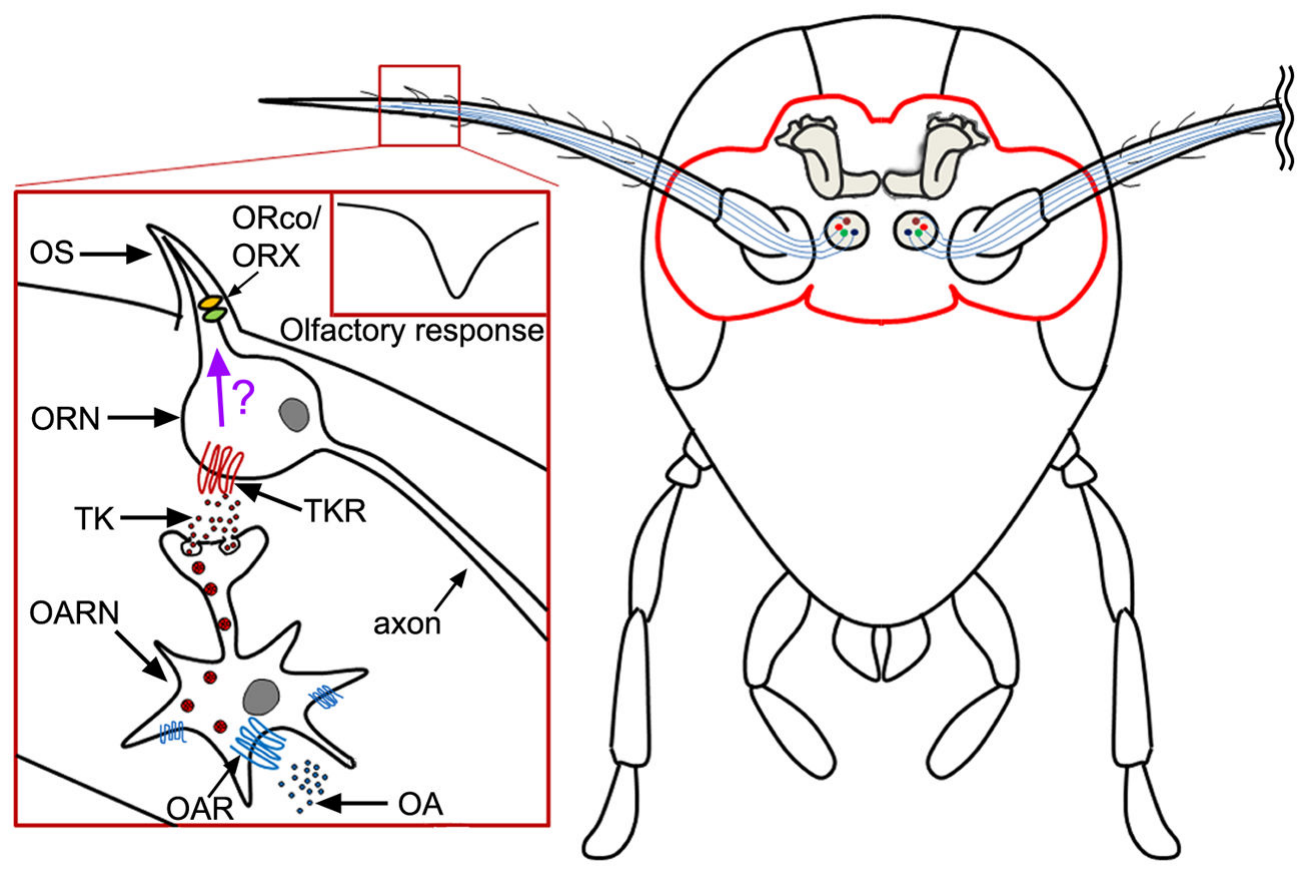
Schematic diagram of olfactory neuromodulation models in the peripheral olfactory organ in the cockroach. Tachykinin is produced from octopamine receptor neurons in antennae. The tachykinin displays the neuro-inhibitory function on olfactory sensitivity changes in the antennal ORNs by unknown mechanisms (arrow with a question mark). OA: Octopamine, OARN: Octopamine receptor neuron, ORN: Odorant receptor neuron, OS: Olfactory sensillum, TK: Tachykinin, TKR: Tachykinin receptor.

Our results have demonstrated that levels of TK regulate the olfactory responses in ORNs of the peripheral olfactory organs. The results suggest that the reduction of olfactory responses by TK reflects either a decrease of the number of responding neurons or efficiency of olfactory signal transduction in ORNs. However, the mechanism by which TK alters olfactory sensitivity in ORNs remains unclear. Further experiments are necessary to demonstrate which olfactory signal transduction components are involved in TK-related signaling in ORNs. Our study has also demonstrated that TK-mediated neural circuits exist in antennal olfactory systems. Although neural circuits associated with sensory facilitation by peptidergic presynaptic modulation has been reported in the ALs of the brain [6,49], our results indicate that TK may have an important role on the fine tuning of peripheral olfactory responses in ORNs, which in turn may subsequently facilitate olfactory perception and recognition. One potential hypothesis is that the release of octopamine into the hemolymph of the antennal heart ultimately activates receptors in antennal neurons which in response release tachykinin. Tachykinin, in turn, could activate its receptors in the ORN to modulate olfactory responses (Figure 6).

A recent study has also shown that the cyclic nucleotides cAMP and cGMP modulate the olfactory sensitivities in ORNs by octopamine and adaptation to pheromone concentration, respectively [50]. Thus, it will be interesting to observe whether there are correlations between TKR components and these cyclic nucleotides in ORNs. Taken together, our study indicates that neuronal networks are present in the peripheral olfactory organ in the cockroach, which is regulated by neuropeptides and biogenic amine systems. 

## Supporting Information

Table S1 **Gene-specific primers used for qRT-PCR and preparation for RNA probes.**
(DOCX)

Table S2 **Active forms of tachykinin peptides in *Periplaneta americana*.**
(DOCX)
