# Supplementary material for: Neuromodulation of Olfactory Sensitivity in the Peripheral Olfactory Organs of the American Cockroach, Periplaneta americana
Source: PLoS One. 2013 Nov 14;8(11):e81361. doi: 10.1371/journal.pone.0081361 (PMC3828268; doi:10.1371/journal.pone.0081361)
Supplement: Table S1 — Gene-specific primers used for qRT-PCR and preparation for RNA probes. (DOCX) [file pone.0081361.s001.docx]

| Application |  | GenBank Access Number | Primer information | Product size (bp) |
| --- | --- | --- | --- | --- |
| RNA probes | *Pa*OA1 | AY333178.1 | Forward primer (FP):  5’-CAACAGCTCCAAGAAGTCCAG-3’  Reverse Primer (RP):  5’-GCTGTCCTCTCCTACCGAGTT-3’ | 754 |
|  | *Pa*TK | AY766012.1 | FP: 5’-CCCCATCACACAACAAGAGTT-3’  RP: 5’-CCCTCCATCTCTGAGTCCTTT-3’; | 615 |
|  | *Pa*TKR | novel gene | FP: 5’-GGGTAGTGAAGATGATGATTGTGGTGG-3’  RP: 5’-TCATCCAACAGTAGATGATGGGATTGTAC-3’  (sequence information on 192 bp of *Pa*TKR: GGGTAGTGAAGATGATGATTGTGGTGGTGTCCATCTTCGCCGTATGCTGGCTGCCGTTTCATATCTACTTCATCGTGACGTCAATCGCGCCAGAAATCACCACAACTAAGTACATCCAGGAGGTGTATCTCGCCATTTACTGGCTGGCCATGTCCAACTCCATGTACAATCCCATCATCTACTGTTGGATGA) | 192 |
|  |  |  |  |  |
| qRT-PCR | *Pa*OA1 | AY333178.1 | FP: 5'-CTCTTCTGGCTGGGCTATTG-3'  RP: 5'-TCCTTGCTAAAGAGGGCGTA-3' |  |
|  | *Pa*TK | AY766012.1 | FP: 5'-GCAAGAAGGCACCATCAGC -3'  RP: 5'-ATGCCCATAAACCCGGAAC-3' |  |
|  | *Pa*TKR | novel gene | FP: 5'- CAAGAGATGGCGAAGCAACAT -3'  RP: 5'-CCCATAAACCCGGAAC-3' |  |
|  | *Pa*Act | AY116670.1 | FP: 5'-GCTATCCAGGCTGTGCTTTC-3'  RP: 5'-ACC GGAATCCAGCACAATAC-3' |  |

Table S1
