# Supplementary figures and images for: Neuromodulation of Olfactory Sensitivity in the Peripheral Olfactory Organs of the American Cockroach, Periplaneta americana
Source: PLoS One. 2013 Nov 14;8(11):e81361. doi: 10.1371/journal.pone.0081361 (PMC3828268; doi:10.1371/journal.pone.0081361)

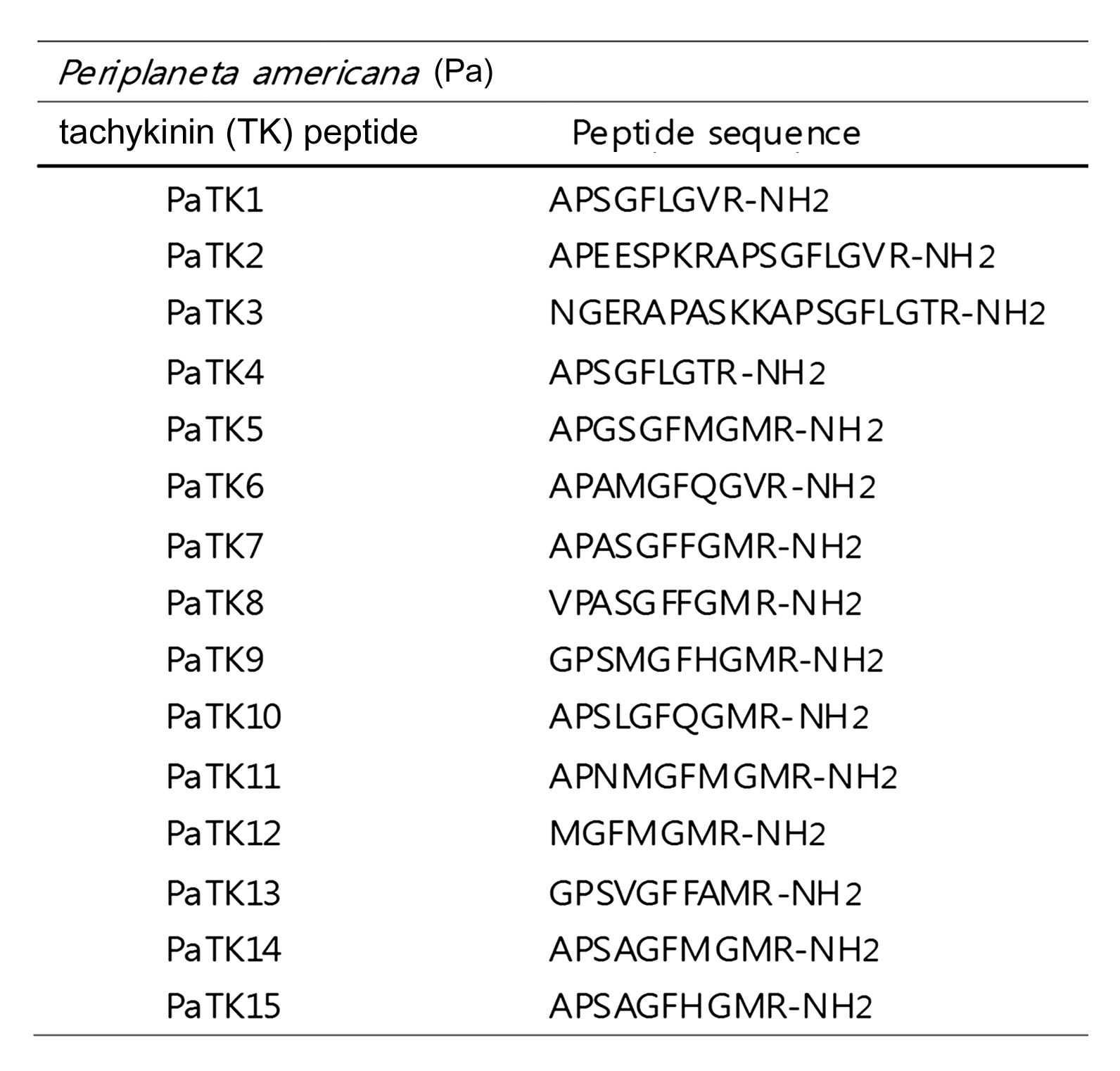
Table S2.

Supplement: Table S2 — Active forms of tachykinin peptides in Periplaneta americana. (DOCX) [file pone.0081361.s002.docx]
